# African Swine Fever Outbreak at a Farm in Central Namibia

**DOI:** 10.1155/2019/3619593

**Published:** 2019-10-29

**Authors:** Alaster Samkange, Borden Mushonga, Douglas Mudimba, Bernard A. Chiwome, Mark Jago, Erick Kandiwa, Alec S. Bishi, Umberto Molini

**Affiliations:** School of Veterinary Medicine, Faculty of Agriculture and Natural Resources, University of Namibia, P. Bag 13301, Pionierspark, Windhoek, Namibia

## Abstract

An outbreak of African swine fever (ASF) occurred at a farm in central Namibia in March 2018. Fourteen pigs died out of a herd of 59 animals over a period of 16 days between the first and sixteenth of March 2018. The clinical signs observed included sternal recumbency, fever, weakness, pain and reluctance to move, hyperemia of the skin and anorexia, followed by death. Necropsy findings included large amounts of unclotted blood in the pleural and peritoneal cavities, diffuse carcass congestion, splenomegaly, consolidation of both lungs, hemorrhagic and frothy airways and trachea, hepatomegaly and congestion, congestion of the gastric mucosa, enlarged and congested kidneys, ecchymotic epicardial, and endocardial hemorrhages, and very enlarged and congested urinary bladder. All the remaining pigs were euthanized, burned, and buried under state veterinary supervision. The authors concluded that the outbreak resulted from indirect transmission of the ASF virus due to lapses in biosecurity measures.

## 1. Introduction

African swine fever (ASF) is an important transboundary hemorrhagic pig disease that is highly contagious, lethal, and has socio-economic implications through its limiting effect on pig production in Africa and elsewhere [1, 2]. The disease is caused by a DNA virus belonging to the only known genus of the *Asfiviridae *family [3]. Infection of pigs by this virus results in strict control measures including culling and quarantine of affected farms and trade embargoes on affected regions or countries.

Clinically, ASF manifests in peracute and acute forms, although subacute and chronic forms may also occur [1, 4–6]. In spite of efforts to develop vaccines to control the disease, success has been elusive [7, 8]. ASF was first reported in 1921 in East Africa from where it spread and became endemic in Southern Africa, [2, 3]. From Southern Africa ASF then spread to other parts of the world including West Africa [8], Europe (Iberian Peninsula countries), Eastern Europe including the Caucasus, and Russian Federation, China, and Brazil [9, 10]. About 23 ASF genotypes have been identified using p72 gene DNA analysis of samples obtained from Africa and only genotype I and genotype II have managed to spread out of Africa [11].

In Namibia, the first confirmed outbreak of ASF occurred in 1932 in Okahandja district [12]. Between 1932 and 1989 a further 41 confirmed ASF outbreaks occurred in the Okahandja, Omaruru, Gobabis, Grootfontein, Swakopmund, and Otjiwarongo districts of the country [13]. ASF is, therefore, endemic in Namibia, particularly in the central areas of the country which are mostly commercial beef farms and where warthogs (*Phacochoerus africanus*) are widespread and roam unrestricted.

At least 3 different cycles have been identified in the transmission of ASF. The first one is an ancient sylvatic cycle involving wild suids, *Ornithodoros moubata *complex ticks, and domestic pigs [1]. The second cycle involves domestic pigs and *Ornithodoros moubata* complex ticks [7]. The third cycle revolves around domestic pigs and their products [14]. The third cycle has evolved concomitantly with the exponential increase in pig production on the African continent gaining prominence in recent times [1, 15]. The epidemiology of ASF is further complicated by the inconsistent immunological responses and socio-economic challenges [1].

The ASF outbreak in this report is part of a low level smoldering series of outbreaks that have continued to be a constant challenge to Namibian veterinary authorities in the central regions of the country. These sporadic outbreaks have been related to the presence, in abundant numbers, of warthogs that freely roam in these regions. This is the first time that ASF has been reported at the farm in question. This case report chronicles the sequence of events that occurred during the ASF outbreak at a farm in the Khomas Region of Namibia. The report further explores possible risk factors and evaluates prevention and control measures for future outbreaks.

## 2. Case Description

An outbreak of ASF occurred at a university teaching farm from the 1^st^ to 16^th^ March 2018. A total of 14 pigs died suddenly after showing some or no acute clinical signs. Prior to the ASF outbreak, the piggery unit at the farm had a total of 59 pigs composed of 3 adult boars, 11 sows, 42 fatteners, and 3 weaners.

**Figure 1:**
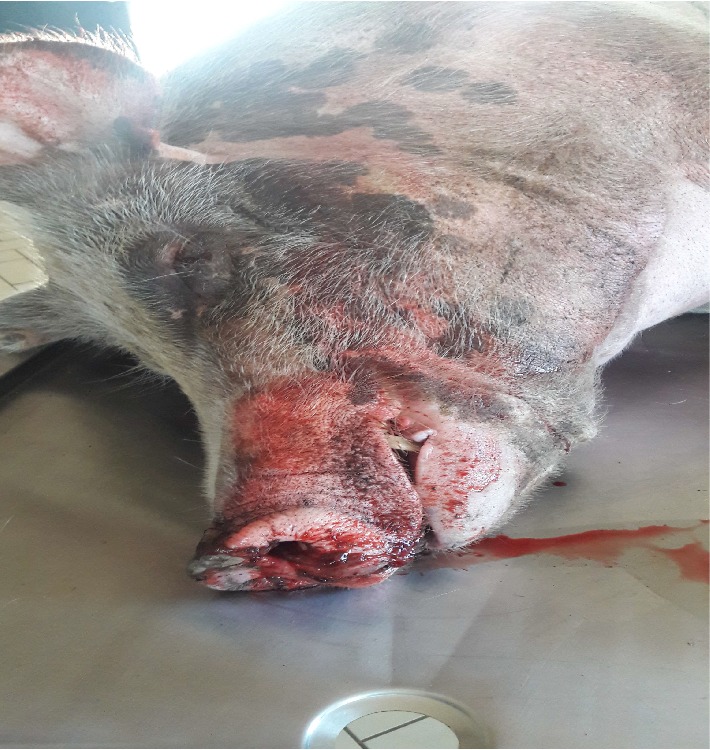
A dead boar with nonclotting blood dripping from the nostrils.

**Figure 2:**
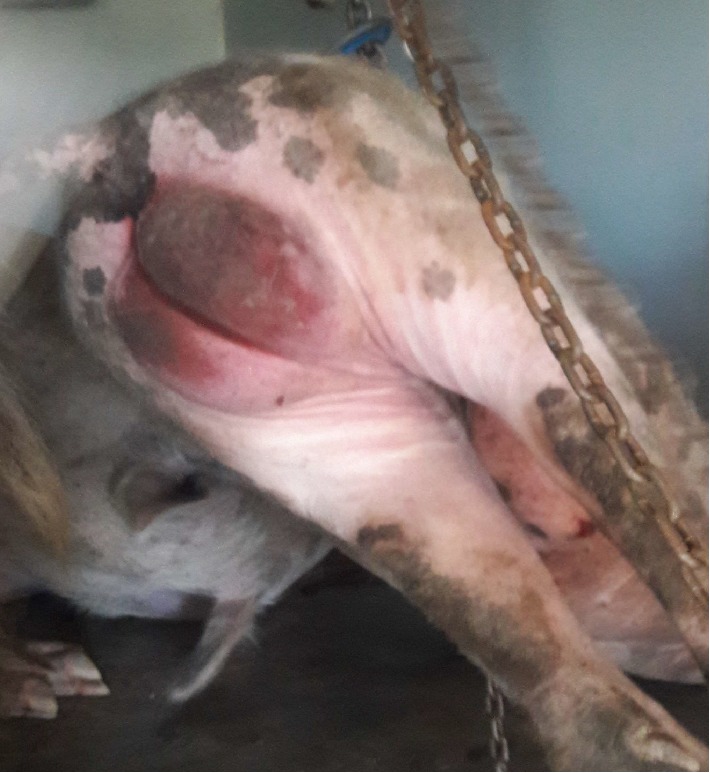
Picture showing hyperemia of the scrotum.

On the 1^st^ March 2018, two adult pigs (a boar and a sow) were found dead without any prior observed clinical signs. Nonclotting blood was found dripping from the nostrils of the two carcasses (Figure 1) as well as hyperemia of the scrotum (Figure 2). To rule out anthrax, blood smears were made and sent to the laboratory for diagnosis and the carcasses were isolated and destroyed intact. The rest of the remaining pigs did not show any clinical signs.

Meanwhile, the remaining herd of 57 pigs was treated with intramuscular injections of Duplocillin® (procaine benzylpenicillin 150 mg/ml, benzathine benzylpenicillin 125 mg/ml) at a dose of 1 ml/20 kg as post-exposure prophylaxis [16]. The blood smears which were sent to the laboratory were stained with M'Fadyean stain and examined, but turned out to be negative for *Bacillus anthracis*.

Four more pigs were found dead on the 7^th^ of March. Upon examination of the rest of the herd, it was discovered that two other pigs, a boar and a sow, were sick. The clinical signs observed included sternal recumbency, weakness, pain and reluctance to move, hyperemia of the skin (especially the scrotum of the boar), and inappetence. The rectal temperatures ranged between 39.5 and 39.8°C and the respiratory rate averaged 10 breaths per minute. The two sick pigs were treated with Synulox RTU® (7.0 mg amoxicillin, 1.75 mg clavulanic acid) at 8.75 mg/kg bodyweight and Finadyne® (flunixin meglumine) at 2.2 mg/kg. However, the animals failed to respond to the treatment and died two days later. Necropsy examinations were performed and fresh samples of the spleen, gastrohepatic lymph nodes, liver, and lungs, on ice, were sent to the Central Veterinary Laboratory for analysis.

**Figure 3:**
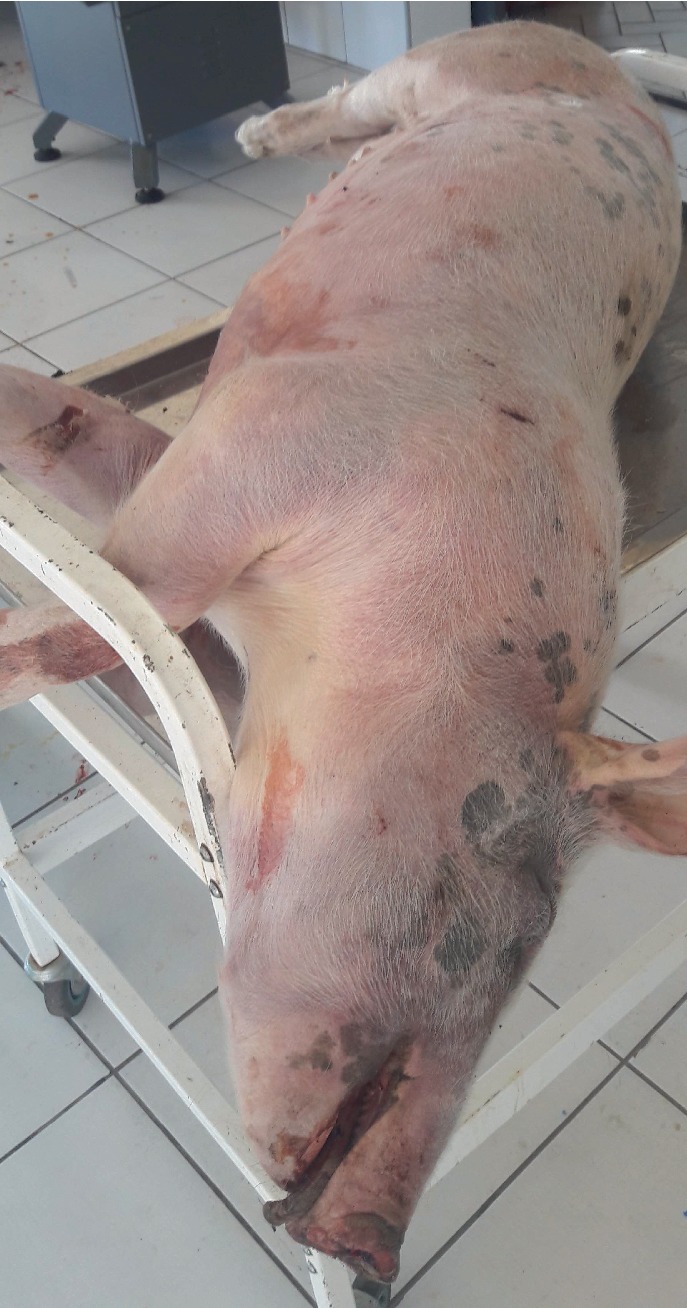
Carcass of a fattener showing large patches of skin hyperemia.

**Figure 4:**
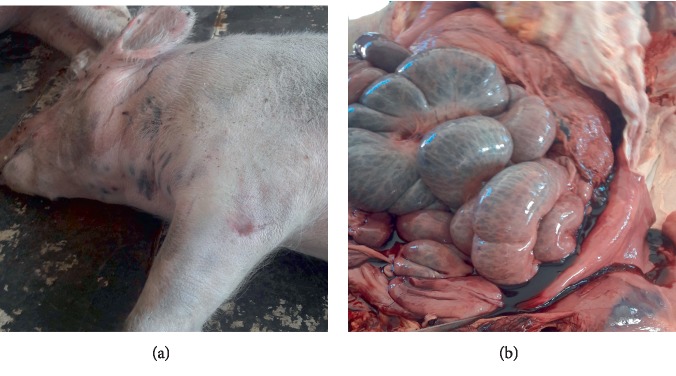
Multifocal ecchymotic skin hemorrhages on the neck (a); frank nonclotting blood in the peritoneal cavity (b).

**Figure 5:**
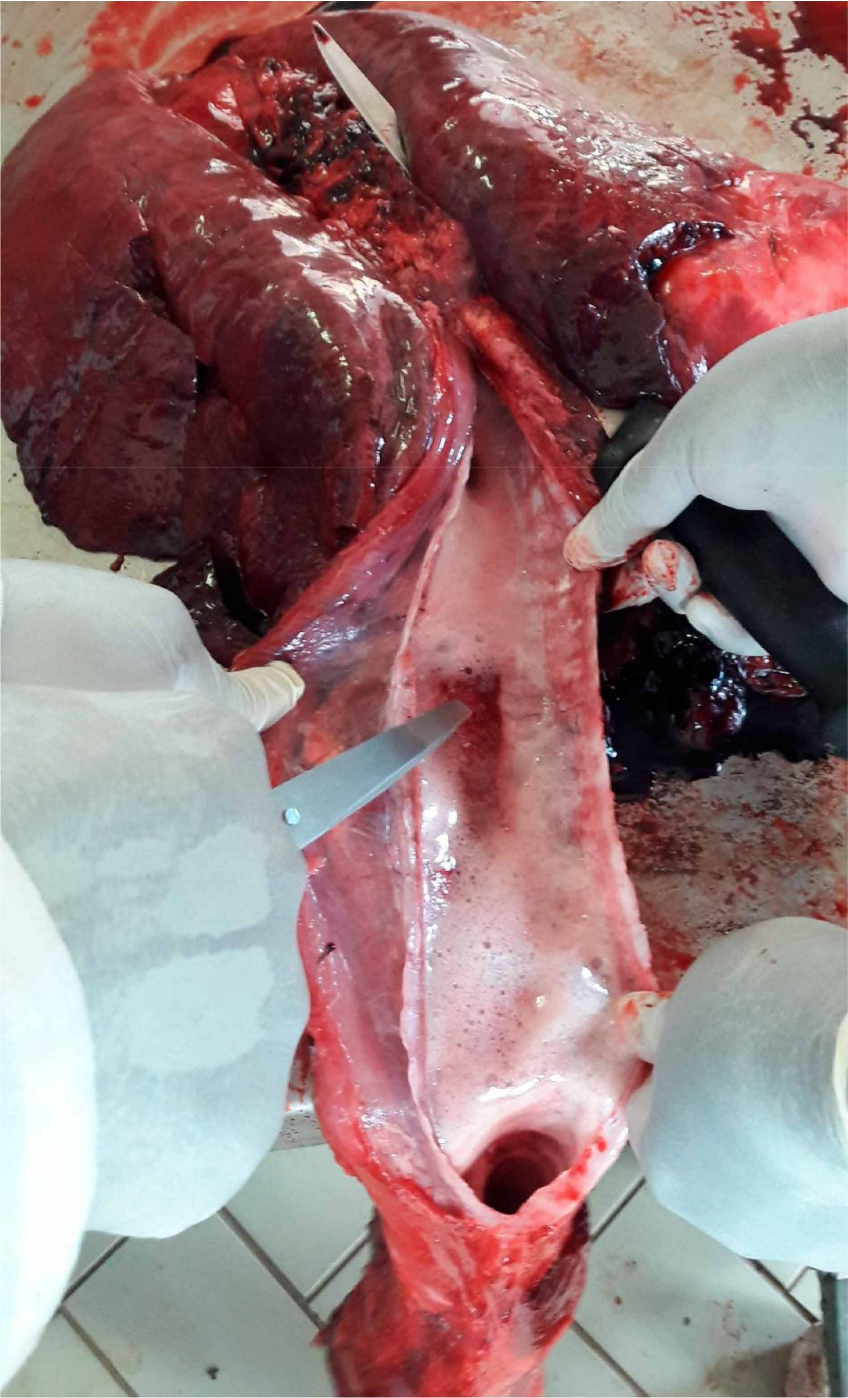
Bilateral red hepatization of the lungs, frothy airways, and congested tracheal mucosa.

**Figure 6:**
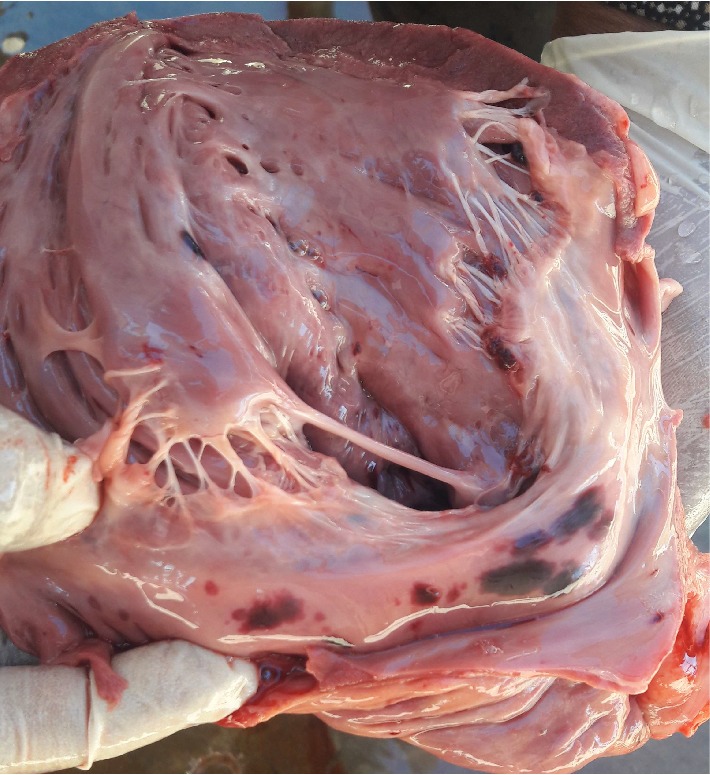
Cut surface of the heart showing ecchymotic endocardial atrial hemorrhages.

**Figure 7:**
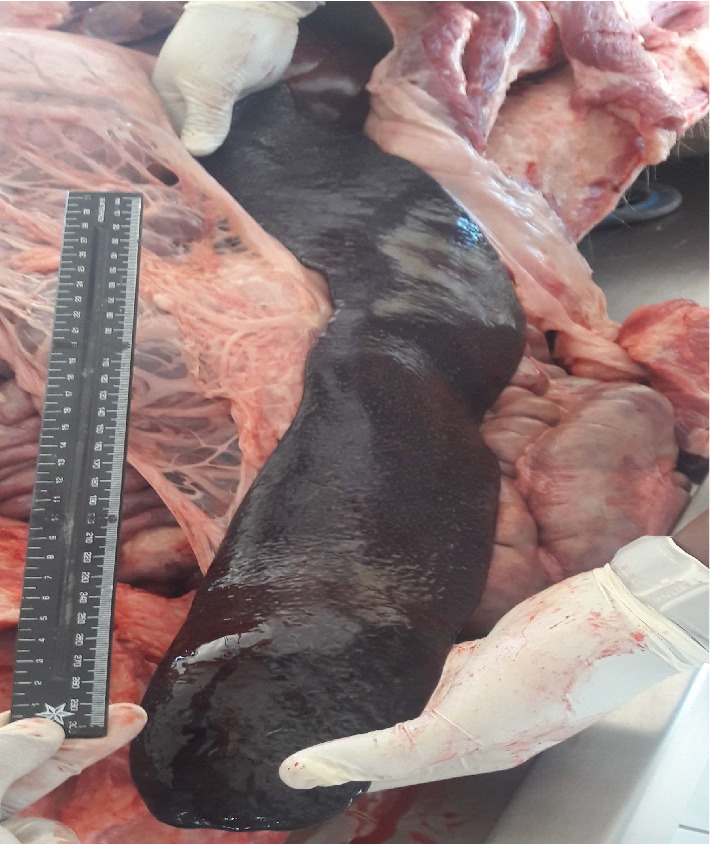
A very enlarged spleen in excess of 30 cm in length.

**Figure 8:**
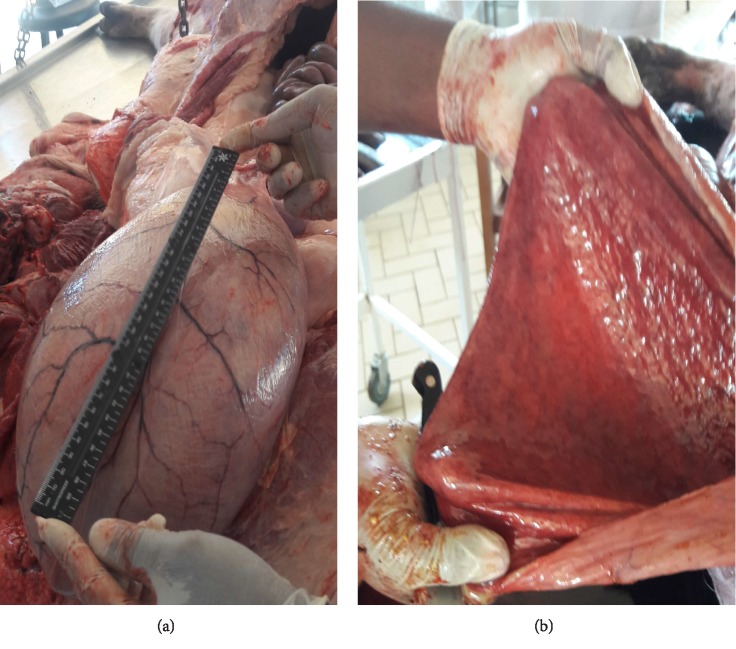
A very distended bladder (a), and congested mucosa of the bladder that was filled with reddish-brown urine (b).

Necropsy****findings included the following: large patches of hyperemia of the skin (Figure 3) in all necropsied pigs, multifocal ecchymotic skin hemorrhages on the neck (Figure 4) in two fatteners, large amounts of unclotted blood in the pleural and peritoneal cavities (Figure 4) in all necropsied pigs, diffuse carcass congestion and splenomegaly (Figure 7) in all pigs, consolidation of both lungs (Figure 5) in adult boars and sows, hemorrhagic and frothy airways including trachea (Figure 5), hepatomegaly and congestion of the liver, congestion of the gastric mucosa, enlarged and congested kidneys, ecchymotic epicardial, and endocardial hemorrhages (Figure 6) in all pigs, very enlarged (approximately 20 cm × 30 cm) and congested urinary bladder (Figure 8) in one boar and one sow.

Total genomic DNA was extracted from the lymph nodes and spleen using a Maxwell®16 Tissue DNA Purification Kit (Promega, Madison, WI, USA) with an elution volume of 300 *μ*l following the manufacturer's instructions. A fragment of the B646L (p72) gene was amplified using a 2X Master Mix (Thermofisher). The primer pair p72-U (forward): 5′- GGCACAAGTTCGGACATGT - 3′ and p72-D (reverse): 5′- GTACTGTAACGCAGCACAG- 3, was used as previously described [17] with the following thermal profile; initial denaturation at 95°C for 5 min and then 40 cycles of denaturation at 95°C for 30 s, annealing at 52°C for 45 s, and elongation at 72°C for 60 s, followed by a final elongation at 72°C for 5 min. All the samples tested negative for African swine fever.

At this stage, the differential diagnoses list still included African swine fever, actinobacillosis (*Actinobacillus suis*, and *Actinobacillus pleuropneumonia*), anthrax, *Mycoplasma suis,* septicemic salmonellosis, and swine erysipelas. Over the following seven days, a total of 10 pigs died peracutely after showing similar or no clinical signs. In addition, some were showing respiratory distress as well as fever (rectal temperatures upwards of 41.5°C). Two of these 10 dead pigs were found moribund and were, therefore, euthanized in accordance with the University of Namibia's School of Veterinary Medicine Animal Welfare Policy Guidelines, using a shotgun and sent to the laboratory as whole carcasses. A standard necropsy was performed and spleen and lymph nodes were collected and tested for ASF using PCR. This time all the samples showed a positivity to ASF producing amplicons of 478 bp.

Upon the confirmation of the diagnosis of African swine fever, the remaining 45 pigs were immediately culled, and the carcasses burned and buried on the farm under the supervision of a state veterinarian. The piggery unit was cleaned and disinfected with peroxygenic acid (Virkon® S), a disinfectant with proven virucidal activity [9]. All exposed equipment, vehicles, and personnel were also disinfected. The piggery was closed down for a mandatory period of three months.

## 3. Discussion

African swine fever is endemic in Namibia, with sporadic outbreaks reported over the past decades [1]. Around the university farm, the presence of wildlife, especially warthogs (*Phacochoerus africanus*), roaming unrestricted on the roadside is a common sight. Warthogs are known asymptomatic carriers of the African swine fever virus [5, 17]. In fact, Namibia is one of the sixteen African countries in which the sylvatic cycle of transmission of ASF is known to occur and warthog contact has either been demonstrated or suspected in previous outbreaks [1].

The presence of *wild suids*, soft ticks of the *Ornithodoros moubaba* complex, and poor biosecurity measures have long been known as risk factors for ASF [8, 14]. Recent studies in Kenya and Nigeria have identified the following as risk factors for ASF: the presence of a slaughter slab within 1-km radius to the piggery, presence of refuse dump sites within 1-km radius to piggery, wearing of piggery unit protective clothing outside the piggery premises, external sourcing of replacement stock, the dry season, feed sources, and tick control [4, 10].

The risk factors for an ASF outbreak existed at the farm prior to the outbreak. For instance, there was an abundance of the warthog in and around the farm. In fact the farm annually harvests and sells game meat (including warthog) to its workers. In addition, the piggery is situated within a kilometer of both a refuse dumping site and a meat processing plant and the northern boundary of the piggery was overgrown with bush and grass. The prior absence of a dedicated piggery attendant resulted in utilization of different workers in turns to feed and take care of the pigs. Some of these workers were known to use the same clothes worn at the piggery at the dairy, poultry, and small ruminant sections. The piggery is also located adjacent to the postmortem hall.

In spite of the presence of biosecurity infrastructure and availability of a biosecurity protocol, implementation of biosecurity measures at the piggery was not strictly adhered to over the years. For instance, access to the piggery was not strictly controlled and was open to visitors; various wildlife like guinea fowls and baboons could enter the premises to feed on pig feed. A study in the Namib Desert of Namibia reported that baboons are often seriously infested by ticks [18] and it is not impossible that baboons could have been a vehicle of infected *Ornithodoros moubata* complex ticks. We speculate that *argasid* ticks could have gained access to the piggery through either guinea fowls or baboons or by crawling from the surrounding bushes.

Although the exact source of the infection could not be identified outright, any of the three cycles namely the wild *suid*-*argasid* tick-domestic pig cycle, the *argasid* tick-pig cycle, or the domestic pig-domestic pig cycle identified in earlier studies [1, 7, 14] could have been responsible for this outbreak.

The authors conclude that this outbreak resulted from indirect transmission of the ASF virus from any of the sources cited above due to laxity in the implementation of biosecurity measures. We, therefore, recommend strict adherence to the biosecurity protocols. In particular, human and animal access to the piggery unit must be controlled, a dedicated and well-trained stockperson is essential; bush and grass clearing of the areas surrounding the piggery and erection of a physical barrier separating the piggery and the necropsy hall would be important biosecurity measures. Strict biosecurity at the piggery unit is of paramount importance if future outbreaks are to be avoided. The practice of harvesting warthogs for meat at the farm should be strictly controlled in order to ensure that anyone who has direct or indirect contact with domestic pigs in the piggery unit does not handle warthog meat.
